# Maternal Factors Pre- and During Delivery Contribute to Gut Microbiota Shaping in Newborns

**DOI:** 10.3389/fcimb.2012.00093

**Published:** 2012-07-05

**Authors:** Giuliano Rigon, Cristina Vallone, Valeria Lucantoni, Fabrizio Signore

**Affiliations:** ^1^Department of Obstetrics and Gynaecology, S. Camillo-Forlanini HospitalRome, Italy

Normally at birth, the human infant gut is sterile, but it becomes fully colonized within a few days with bacteria from the mother and the environment (Salminen and Isolauri, 2008).

An altered gut microbiota composition has been associated with attenuated immune responses to inflammation in experimental models and humans (Fanaro et al., 2003; Ly et al., 2011; Vebø et al., 2011).

The pioneer microbiota of the neonate may affect future actions of the immune system (Conroy and Walker, 2008; Karlsson et al., 2011; Vael et al., 2011).

The relation between the neonatal gut microbiota and the development of allergic diseases and obesity has led to several clinical trials of probiotics (live bacteria given orally that allow for intestinal colonization) in human subjects both during pregnancy or in the neonatal period. Probiotic trials thus far have failed to show a consistent preventive effect (Litonjua, 2012).

Many factors contribute to the shaping of this complex ecosystem, and all must be taken into account. Maternal gut microbiota is a factor in neonatal colonization. Reduced concentrations of *Bifidobacterium* and *Bacteroides* and increased numbers of *Staphylococcus*, Enterobacteriaceae were detected in overweight compared with normal-weight pregnant women (Santacruz et al., 2010).

Increased Enterobacteriaceae numbers were related to increased ferritin and reduced transferrin, while *Bacteroides* numbers were related to increased HDL-cholesterol and folic acid levels (Santacruz et al., 2010; Table 1).

**Table 1:** **Effects of neonatal physiological and pathological conditions on gut microbiota onset and distribution**.

Bacterial species	Prevalence/abundance	Decrease/abatement	Physiological conditions	Pathological conditions	Reference
*Escherichia coli*	+++		Increased ferritin/reduced transferrin		Santacruz et al. (2010)
*Bacteroides* spp.	+++		Increased HDL-cholesterol/folic acid		Santacruz et al. (2010)
*Staphylococcus* spp., *E. coli*	+++			Overweight pregnant women	Santacruz et al. (2010)
*Bifidobacterium* spp., *Bacteroides* spp.		+++		Overweight pregnant women	Santacruz et al. (2010)
*Bifidobacterium* spp., *Lactobacillus* spp.	+++		Baby breast feeding		Fanaro et al. (2003), Rinne et al. (2006)
*Clostridium, Bacteroides, Enterococcus* spp.		+++	Baby breast feeding		Fallani et al. (2010), Penders et al. (2006)
*Bacteroides* spp., *Atopobium* spp., *Bifidobacterium* spp.		+++	Perinatal antibiotics treatment		Penders et al. (2006)
*Bacteroides* spp.		+++		Mouse obesity	Ley et al. (2005)
*Firmicutes*	+++			Mouse obesity	Ley et al. (2005)
*Clostridium* spp., *Bacteroides* spp., *Staphylococcus* spp., *Akkermansia* spp.	+++			Overweight pregnant women	Collado et al. (2010)
*Lactobacillus* spp*., Bifidobacterium* spp.		+++		Extremely low-birth-weight infants	Fanaro et al. (2003), Rougé et al. (2010), Dai and Walker (1999)
*Clostridium* spp., *Bacillus* spp.	+++		Vaginal delivery		Penders et al. (2006), Lif Holgerson et al. (2011), Huurre et al. (2008)
*Bacteroides* spp.*, Atopobium* spp., *Bifidobacterium* spp.		+++		Cesarean section	Fallani et al. (2010)
*Bifidobacterium* spp.	+++		Probiotic administration during pregnancy		Gueimonde et al. (2006)
*Lactobacillus* spp., *Enterococcus* spp., *Clostridium* spp.		+++	Probiotic administration during pregnancy		Rinne et al. (2006)

Population related factors are significant (Bäckhed, 2011). Karlsson et al. (2011) observed *Lactobacillus* in all neonates, other bacterial groups were detected only in 14–30% of the subjects (*Bifidobacterium*, *Enterococcus*, and the *Bacteroides fragilis* group). Fallani compared neonatal fecal samples from Sweden, Scotland, Germany, Italy, and Spain. *Bifidobacterium* genus was predominant (40% average proportion of total detectable bacteria), followed by *Bacteroides* (11.4%) and Enterobacteria (7.5%; Fallani et al., 2010). Differences in colonization pattern can be observed between infants in industrialized and developing countries (Adlerberth, 2008). Siblings increase the numbers of Bifidobacteria, while pets and country residence show no significance (Penders et al., 2006). Dominguez observed a neonatal colonization corresponding to maternal skin population in case of cesarean section and coincident with maternal vaginal flora in case of vaginal delivery (Dominguez-Bello et al., 2010).

Breastfeeding is a significant factor in the determination of neonatal gut microbiota. During lactation, cells from gut-associated lymphoid tissue travel to the breast via the lymphatics and peripheral blood (Donnet-Hughes et al., 2010).

Breast milk gives a flora rich in *Bifidobacterium* spp. Other obligate anaerobes, such as *Clostridium* spp. and *Bacteroides* spp., are more rarely isolated and also Enterobacteria and Enterococci are relatively few. Formula-fed babies are often colonized by other anaerobes in addition to Bifidobacteria and by facultatively anaerobic bacteria; the development of a “*Bifidus* flora” is unusual (Fanaro et al., 2003). Breastfeeding leads to higher *Lactobacillus* and lower count of *E. coli*, *Clostridium difficile*, *B. fragilis* (Penders et al., 2006; Fallani et al., 2010). After delivery, breastfeeding continues to enhance the original inoculum by specific lactic acid bacteria and Bifidobacteria and bacteria from the mother's skin enabling the infant gut microbiota to be dominated by Bifidobacteria. Modifying this exposure can take place by probiotic bacteria when breastfeeding is not possible (Conroy and Walker, 2008; Salminen and Isolauri, 2008). Fecal *Bifidobacterium* and *Lactobacillus*/*Enterococcus* spp. counts were higher in breastfed than formula-fed infants at 6 months (Rinne et al., 2006; Table 1). Maternal and neonatal medical treatment is an issue. Newborns from mothers treated with antibiotics perinatally had lower proportions of *Bacteroides* and members of the *Atopobium* cluster. Antibiotics lower the count of Bifidobacteria and *B. fragilis* group, according to Penders et al. (2006). Gut microbiota is influenced by perinatal conditions. Ley et al. (2005) found that obese pregnant mice have a 50% reduction in *Bacteroidetes* and a proportional increase in Firmicutes compared to normal controls on the same diet. One mechanism here could lie in the ability of specific gut microbes to induce excessive energy harvests (Collado et al., 2008). Overweight women show increases of *Clostridium*, *Bacteroides*, *Staphylococcus*, and *Akkermansia* during pregnancy according to the author. Their infants’ fecal microbial composition was related to the weight and weight gain of their mothers during pregnancy (Collado et al., 2010; Table 1).

In extremely low-birth-weight infants characterized by antibiotic therapy, parenteral nutrition, delayed oral feedings, and intubation the gut is colonized by a small number of bacterial species; *Lactobacillus* and *Bifidobacterium* spp. are seldom identified (Fanaro et al., 2003). Rougé et al. (2010) indicated that the gastrointestinal tract of preterm infants, born less than 33 weeks, has a low biodiversity. According to Dai and Walker (1999), premature infants requiring intensive care acquire intestinal organisms slowly, and the establishment of bifidobacterial flora is retarded. The aberrant colonization of the premature infant may contribute to the development of necrotizing enterocolitis.

Neonatal gut is related to mode of delivery. In infants born by cesarean section (C-section) the establishment of a stable flora is delayed (Fanaro et al., 2003). Significantly more bacterial taxa were detected in the infants delivered vaginally (79 species/species clusters) compared with infants delivered by C-section (54 species/species clusters; Lif Holgerson et al., 2011). Newborns delivered by C-section had lower proportions of *Bacteroides* and members of the *Atopobium* cluster (Fallani et al., 2010). Infants delivered by C-section had fewer bifidobacteria at an early age and were shown to mount a stronger humoral immune response (Huurre et al., 2008). At 1 month of age, the total gut bacterial cell counts per 1 g feces were higher in vaginally delivered infants. This distinction was mainly due to the greater number of Bifidobacteria in vaginally delivered infants. During the first year of life, the total number of immunoglobulin (Ig) A, IgG-, and IgM-secreting cells was lower in infants born by vaginal delivery than in those born by C-section, possibly reflecting different antigen exposure (Huurre et al., 2008).

Dominguez observed a neonatal colonization corresponding to maternal skin population in case of c-section and coincident with maternal vaginal flora in case of vaginal delivery (Dominguez-Bello et al., 2010). An extensive Netherlands study shows conclusively that vaginal delivery brings on a faster colonization by all species, mostly Bifidobacteria, with high *B. fragilis* and low *C. difficile* counts (Table 1). High *Clostridium* counts were associated with clinical complications and hospital admittance (Penders et al., 2006). Most significantly things change with age. The bacterial flora is usually heterogeneous during the first days of life, independently of feeding habits. After the first week of life, a stable bacterial flora is usually established (Fanaro et al., 2003).

The first bacteria to establish in the neonatal gut are usually aerobic or facultative anaerobic bacteria, like Enterobacteria, Enterococci, and Staphylococci. During their growth, they consume oxygen and change the intestinal milieu making it suitable for the proliferation of anaerobic bacteria. *Bifidobacterium*, *Clostridium*, and *Bacteroides* are among the first anaerobes establishing in the microbiota. As more oxygen-sensitive species establish and the complexity of the microbiota increases, the population sizes of aerobic and facultative bacteria decline (Adlerberth, 2008).

Vebo showed a decrease in Staphylococci from 10 days to 4 months and a peak of Bifidobacteria and *Bacteroides* at 4 months (Gueimonde et al., 2006; Vebø et al., 2011). Clinical effects of an altered neonatal colonization have been noticed. In the past 20–30 years, the prevalence of atopic diseases, particularly among children in the Western world, has increased. It has been suggested that Western lifestyle may have reduced the overall exposure to microbial stimulation early in life (øien et al., 2006).

The pioneer microbiota of the neonate may affect future actions of the immune system (Karlsson et al., 2011). A close relationship between allergic sensitization and the development of the intestinal microflora may occur in infancy. Intestinal micro-organisms could down-regulate the allergic inflammation by counterbalancing type 2 T-helper cell responses and by enhancing antigen exclusion through an immunoglobulin Ig-A response (Kirjavainen and Gibson, 1999). According to Vael et al. (2011), early colonization by *Clostridium coccoides* or *B. fragilis* could lead to asthma in later life.

Altered gut colonization could lead to obesity in later life. Germ-free mice are protected against developing diet-induced obesity. The gut microbiota affects expression of secreted proteins in the gut, which modulate lipid metabolism in peripheral organs and is a source of pro-inflammatory molecules that augment adipose inflammation and macrophage recruitment by signaling through the innate immune system (Bäckhed, 2011). An intriguing observation is that neonates treated with antibiotics during the first 6 months of life had an increased risk of overweight among children of normal-weight mothers (OR: 1.54, 95% CI: 1.09–2.17) with a decreased risk of overweight among children of overweight mothers (Ajslev et al., 2011). Trials with probiotic precursors administered to the mother have been reported. Maternal administration of *Lactobacillus rhamnosus* GG (L-GG, ATCC 53103) during late pregnancy promotes a Bifidobacteria profile of infant gut microbiota, similar to that of a healthy breastfed infant. Microbial diversity in neonatal gut microbiota was not influenced by this probiotic administration at 1 week postpartum (Ismail et al., 2012). In a prospective randomized study Gueimonde et al. (2006) showed that maternal *L. rhamnosus* administration during late pregnancy is associated in the neonates at 5 days of age with a higher occurrence of *Bifidobacterium brevis* and lower of *Bifidobacterium adolescentis*. Probiotic supplementation has been tried in newborns. One-hundred thirty-two neonates were randomized in a placebo group and the others were treated with *L. rhamnosus*. For 6 months after delivery, mothers had been treated prenatally for 6 months in the treatment group. At 6 months, there were less Clostridia in feces in the placebo compared with the probiotic group (*P* = 0.026), whereas after long-term follow-up at 2 years, there were less Lactobacilli/Enterococci and Clostridia in feces in the probiotic group than in the placebo group (Rinne et al., 2006; Table 1).

Rinne showed in another randomized trial of neonatal *L. rhamnosus* administration that at 3 months IgG-secreting cells in breastfed infants supplemented with probiotics was higher (Rinne et al., 2005). Chierici underlined the importance of a probiotic diet with bifidogenic activity of non-digestible but fermentable carbohydrates (Chierici et al., 2003).

In conclusion, we infer that many observations indicate the significance of bacterial neonatal colonization of the gut. A tighter control of factors influencing this phenomenon is warranted if results of preventive or therapeutic measures, or effects of maternal, or perinatal conditions is to be identified.
